# Emerging Potential of Plant Virus Nanoparticles (PVNPs) in Anticancer Immunotherapies

**DOI:** 10.33696/cancerimmunol.4.061

**Published:** 2022

**Authors:** Mehdi Shahgolzari, Steven Fiering

**Affiliations:** 1 Department of Advanced Sciences and Technologies in Medicine, School of Medicine, North Khorasan University of Medical Sciences, Bojnurd, Iran; 2 Department of Microbiology and Immunology, Dartmouth Geisel School of Medicine, Hanover, NH, United States; 3 Norris Cotton Cancer Center, Dartmouth Geisel School of Medicine and Dartmouth-Hitchcock Medical Center, Lebanon, NH, United States

**Keywords:** Immunostimulatory reagent, Nanoparticles, Neoantigens, Pathogen-associated molecular patterns, Virus-like particle

## Abstract

Cancer immunotherapies using plant virus nanoparticles (PVNPs) have achieved considerable success in preclinical studies. PVNP based nanoplatforms can be endogenous immune adjuvants and act as nanocarriers that stabilize and deliver cancer antigens and exogenous immune adjuvants. Although they do not infect mammalian cells, PVNPs are viruses and they are variably recognized by pathogen pattern recognition receptors (PRR), activate innate immune cells including antigen-presenting cells (APCs), and increase the expression of costimulatory molecules. Novel immunotherapy strategies use them as *in situ* vaccines (ISV) that can effectively inhibit tumor growth after intratumoral administration and generate expanded systemic antitumor immunity. PVNPs combined with other tumor immunotherapeutic options and other modalities of oncotherapy can improve both local and systemic anti-tumor immune responses. While not yet in clinical trials in humans, there is accelerating interest and research of the potential of PVNPs for ISV immune therapy for cancer. Thus, antitumor efficacy of PVNPs by themselves, or loaded with soluble toll-like receptor (TLR) agonists and/or cancer antigens, will likely enter human trials over the next few years and potentially contribute to next-generation antitumor immune-based therapies.

## Introduction

Plant virus nanoparticles (PVNPs) are increasingly recognized and studied for use in biomedical applications. PVNPs include plant virions with self-assembled capsid protein coats (PC) that encapsulate the virus genome, and virus-like particles (VLPs), a capsid without the viral genome. Both virions and VLPs are noninfectious for mammals and do not replicate within tumor cells or other mammalian cells [1]. Virions can infect appropriate plant hosts while VLPs cannot. PVNPs are considered nanoparticles because of their nanoscale size, which are generally in the range of 20–300 nm. PVNPs are useful in cancer therapy because they not only can act as vehicle for delivery of anticancer agents but some have strong immunostimulatory properties and support antitumor immunity [2]. The hollow structure and external protein surface of PVNP capsids allows loading of cancer therapy agents by genetic and/or physico-chemical engineering. In particular, PVNPs can be engineered to display antigen/epitopes, and/or encapsulate immune agents that further modulate immune cells and enable anti-tumor immunization [2,3]. These properties have been exploited for the generation of vaccines against chronic inflammatory conditions, neurodegeneration, allergies, cancer, bacteria, viruses (including COVID-19) and treatment of autoimmune diseases [4–6].

PVNPs are promising candidates for the development of next generation anticancer immunotherapies. When applied as nanocarriers, the PVNP formulations can go beyond the natural immune stimulatory properties of many PVNPs and deliver cancer antigens or exogenous adjuvants. When applied as immunostimulatory reagents, PVNPs can reprogram the tumor microenvironment from immunosuppressive to more immunostimulatory, which generates local and systemic anti-tumor immunity [7].

## Delivery Strategies of PVNPs for Cancer Immunotherapy

PVNPs have been explored as a unique class of nanoparticles for drug delivery, imaging, immunotherapy, and theranostic applications [8]. PVNPs hold promise for cancer immunotherapies via delivery of cancer antigens, adjuvants, and modulation of the immune-suppressive tumor microenvironment (TME) [7]. Delivery strategies of PVNPs for cancer immunotherapy have focused on systemic administration, e.g. intraperitoneal (I.P.), intravenous (I.V.), or intratracheal routes [9–11] that often have challenging pharmacokinetics with insufficient delivery to tumors. Systemic delivery of PVNP cancer therapies has struggled with the reality that phagocytes avidly ingest PVNPs with which they interact [12–15]. Most systemically IV delivered nanoparticles are sequestered in phagocytes in liver or spleen and do not get to the tumor [13–16]. This show that routes of administration can influence the therapeutic efficacy of delivery technologies. A relevant strategy for PVNPs and other nanoparticles is direct intratumoral delivery, as is approved by the FDA for the oncolytic virus T-VEC [17]. One concept relevant to intratumoral immunotherapy is “*in situ* vaccination” (ISV). This simple strategy relies on three basic concepts in all cancer immunotherapy: 1) tumors are recognized by the immune system; 2) clinically identified tumors are protected from the immune system by local tumor-generated immune suppression; 3) any clinically effective immunotherapy must overcome this immune suppression.

Every vaccine has two components, antigen to be recognized by the immune system and immune adjuvant to stimulate response against microorganisms or cells that carry the antigen [7]. Tumor antigens are highly variable and generally quite specific for each patient, particularly patient-specific are “neoantigens” that are generated by mutations that are randomly generated in many tumors. ISV directly applies an immune stimulating adjuvant to the tumor, and depends on the antigens that are naturally within the tumor as the antigen source for the vaccine [18]. The goal is to disrupt the local immunosuppression, activate the myeloid cells within the tumor and thus stimulate an effective innate immune response against the treated tumor, and activate tumor-recognizing effector T cells that then circulate and increase the immune pressure on other metastatic tumors that were not directly treated [11]. When optimally done, this improved systemic antitumor immunity by itself or with other systemic immunotherapies, like checkpoint blockade, reduces or eliminates the metastatic disease that causes the preponderance of patient morbidity and mortality from most types of solid tumors.

*In situ* vaccination is not focused on local control of treated tumors, although it can accomplish that. Local control with surgery and/or radiation or other energy delivery like heat or high intensity focused ultrasound is generally quite successful at local tumor elimination. The true value of ISV is in generating expanded systemic antitumor immune responses. While PVNPs can be utilized as systemically-delivered therapy, and some examples are noted below, *in situ* vaccination using intratumoral delivery of PVNPs is the focus of most current interest.

As immunotherapy, ISV has advantages: rapid delivery since it is not patient specific and causes rapid reprogramming of the TME, so could be done between pathologic diagnosis and surgery; less expense since the amount of reagents used are much lower than what is needed for systemic immunotherapy; generally safe because again, while the reagents are immunostimulatory they are administered at low levels systemically, so the inflammatory response tends to be local. While it is true that different tumor locations vary in their ease of ISV delivery, it is also true that surgeons and interventional radiologists can safely inject tumors found in almost any anatomical location.

The importance of uniform intratumoral distribution of PVNP during treatment application is assumed but not tested, and may not be important for optimal efficacy. It is difficult to obtain uniform distribution with needle injections due to intratumoral heterogenicity and the generally high interstitial fluid pressure in tumors [19–21]. New administration options for localized immunotherapy are being developed, including passive and active microneedle patches and implantable scaffolds that degrade and release reagents [21,22]. Immunotherapeutic vaccines are generally administered multiple times; however, as with any treatment, each required treatment increases expense and has reduced compliance from patients. PVNPs of CPMV have also been applied as a slow-release formulation by forming aggregates with polyamidoamine generation 4 dendrimers (CPMV-G4) [8,39]. Comparing administration techniques with associated results should enable a better understanding of how administration affects ISV responses.

For PVNPs and other immune stimulating nanoparticles, the tendency to be ingested by phagocytes makes them more valuable for ISV [7]. Phagocytes such as monocytes, dendritic cells, macrophages, and neutrophils are almost always found in tumors where they have immune suppressive phenotypes. The tendency of phagocytes to ingest nanoparticles focuses the immunostimulatory properties on those cells, where that immune stimulation can change their phenotype from suppressor cells to myeloid effector cells that directly attack the tumor, and antigen presenting cells that ingest tumor antigens, travel to the draining lymph nodes and present antigen to activate antitumor T cell responses [23]. These stimulated tumor antigen-recognizing T cells expand in numbers and circulate to find and attack other tumors, generating systemic antitumor immunity and eventually immune memory.

## Induction of Innate Immune Responses by PVNPs

Innate immune cells are activated primarily by exogenous pathogen-associated molecular patterns (PAMPs) or endogenous damage-associated molecular patterns (DAMPs), that stimulate pattern recognition receptors (PRRs) [24,25]. PRRs include membrane, endosomal, cytoplasmic and soluble PRRs that upon interaction with their ligands alter gene expression of the cell [25,26]. One well-studied class of PRRs are the toll-like receptors (TLRs) that primarily recognize PAMPs. TLRs are localized on the cell surface (e.g. TLR1/2/4/5/6) and within endosomes (e.g. TLR3/7/8/9). Binding of their ligands activates signaling pathways that in turn activate transcription factors such as nuclear factor-kappa B (NF-κB) and IFN regulatory factors (IRFs) which stimulate transcription and secretion of inflammatory cytokines, chemokines and type I IFNs [27]. A variety of lab-generated molecules have been developed as TLRs agonists such as polyinosinic: polycytidylic acid (poly (I: C)) for TLR3, imiquimod (R837) and resiquimod (R848) for TLR7/8 and CpG oligodeoxynucleotides (ODNs) for TLR9 [28]. In general, the endocytic TLRs recognize nucleic acids and the surface TLRs recognize proteins or other complex molecules, like lipopolysaccharides.

**Figure 1:**
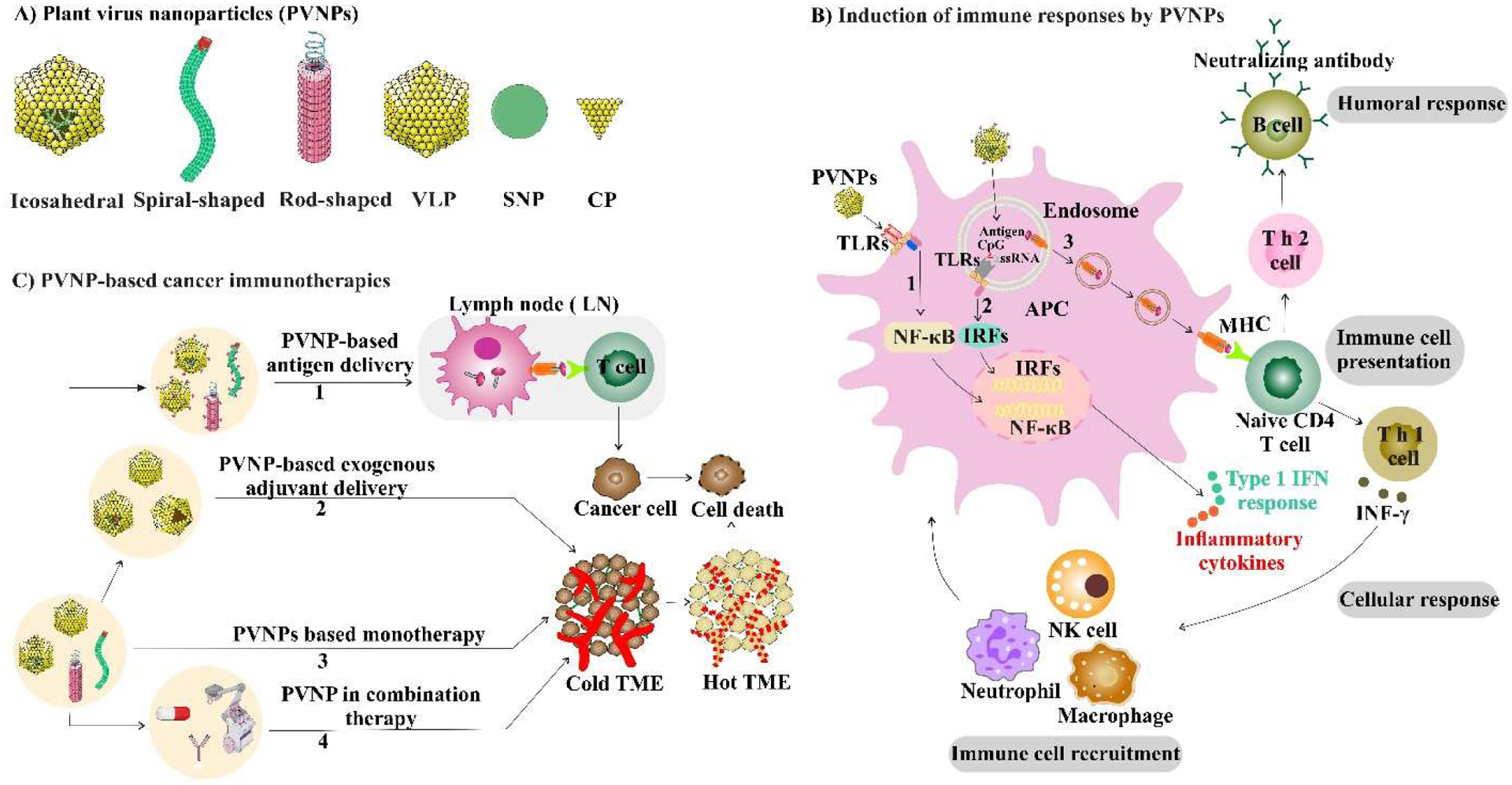
Plant virus nanoparticles (PVNPs) and how they can regulate antitumor immunity. A) PVNPs with various shapes and derivations (VLP, SNP, CP). B) Activation of APCs by PVNPs via surface Toll-like receptors (TLRs) (path 1), or endocytosis of PVNPs, PVNPs-loaded TLR agonists (path 2) or PVNPs-loaded cancer antigens (path 3), and the expression of antiviral, proinflammatory cytokines, induce humoral and cellular responses. C) PVNP -based cancer immunotherapies, 1) cancer antigen conjugated to PVNPs and resulted in higher responses against that antigen, 2) stimulatory agents is encapsulated by PVNPs and significantly enhances the efficacy. 3) Monotherapy; *in situ* vaccination of PVNPs overcomes the local immunosuppression and stimulates a potent anti-tumor response. 4) PVNP based monotherapy in combined with other therapies (blocking or agonist antibodies, radiation therapy, and chemotherapy).

Many PVNPs are recognized by innate immune cells as non-self, although with varying levels of stimulatory responses. This recognition is perhaps not surprising, since they are viruses, although mechanistically it is minimally studied. Questions such as why some PVNPs are strongly immune stimulatory while other apparently quite similar PVNPs have minimal recognition are not yet well understood. Immune recognition of PVNP depends on various characteristics such as particulate nature, repetitive protein structure, and nucleic acid content [29]. It was shown that PVNP capsids with an organized regular spatial structure are more immunogenic than disassembled capsids and their associated coat proteins (CP) [11,29]. The nanoparticle nature of PVNPs stimulates phagocytosis by various phagocytic cells, including antigen presenting cells (APC) [29,30] but tendency for phagocytic uptake also varies for unclear reasons. A variety of PVNP-related structures including native virions, VLP, spherical nanoparticle (SNP) and CP of some PVNP (Figure 1A) can serve as adjuvants and induce APC activation and expression of antiviral and proinflammatory cytokines [7,29,31]. PVNPs can activate surface TLRs, while endocytosis of PVNPs containing nucleic acid by APCs contribute to an immune response by stimulating endosomal TLRs (Figure 1B) [7,32,33]. One aspect of note is that unlike most animal viruses, plant viruses are not enveloped, which may facilitate recognition by some PRR receptors, such as TLR4, that generally do not recognize enveloped mammalian viruses.

Basic mechanisms of PVNP ISV in treating solid tumors can be summarized by a series of mechanistic steps: 1) the PVNP is delivered directly into the tumor; 2) the PVNP is taken up by various innate immune cells, particularly phagocytes, which become activated; 3) the activated innate cells release cytokines and chemokines that attract greater numbers of activated innate cells to infiltrate the tumor and attack tumor cells 4) T-lymphocytes are presented antigen by the activated APCs in tumor draining lymph nodes, become activated, are attracted to the tumor and attack tumor cells carrying their cognate antigens leading to tumor lysis, 5) activated T-lymphocytes travel systemically and attack metastatic tumors throughout the body [8].

## Multifunctional PVNPs for Cancer Immunotherapies

Antitumor immunity efficacy of PVNP is achieved via disruption of the immunosuppressive TME because of immune adjuvant activity with or without cancer antigen delivery [7]. PVNP-based cancer vaccines can be used to induce tumor associated antigen-specific immune responses by targeting tumor cells expressing cancer-driving receptors. Recent studies reported on human epidermal growth factor receptor-2 (HER2)-specific cancer vaccines using different PVNPs such as icosahedral cowpea mosaic virus (CPMV), cowpea chlorotic mottle virus (CCMV), Sesbania mosaic virus (SeMV), Physalis mottle virus (PhMV) and filamentous potato virus X (PVX) [3,34–36]. CPMV has also been used for delivering immunogenic cancer-associated testis antigen NY-ESO-1 [37].

While these PVNPs vary in ability to serve as immune adjuvants, they differ in other properties, including ability to be manipulated to carry other molecules. PVNPs can be modified to improve antitumor efficacy via encapsulation of a soluble adjuvant so that it has nanoparticle properties, which include preferential ingestion by phagocytes. For example, loading CCMV with CpG oligonucleotides promotes activation of tumor associated macrophages (TAMs) *ex vivo* and *in vivo* [38]. While *in-situ* vaccination using PVNPs produces significant treatment efficacy as cancer therapy, effects of systemic administration are weak because they are sequestered away from the tumor by the mononuclear phagocyte system [13–15].

One outcome of repeated delivery of foreign proteins like PVNPs is generation of antibodies against the proteins. This will “neutralize” oncolytic viruses since their impact depends on productive infection of the mammalian cells. However, since PVNPs do not infect mammalian cells, they are not “neutralized” by anti-PVNP antibodies. Interestingly, the presence of anti-CPMV antibodies not only did not inhibit the efficacy of CPMV-based ISV, but rather improved the antitumor efficacy [14]. While not extensively studied, this makes sense since antibody coating (opsonization) of a nanoparticle is expected to increase both ingestion by phagocytes as well as stimulation and activation of the ingesting phagocytes.

### CPMV based monotherapy

CPMV has the been the most well-studied PVNP and is a more potent immune stimulatory reagent than most PVNPs, for reasons that are not fully understood [21]. CPMV is an icosahedral single-stranded RNA (ssRNA) virus that is rapidly ingested by phagocytes *in vitro* and *in vivo* [40]. CPMV is recognized by MyD88-dependent toll-like receptors (TLRs). The assembled capsid is recognized by TLR2 and TLR4 and the encapsidated ssRNA is recognized by TLR7 which uniquely induces secretion of type I interferons (IFNs), and contributes to CPMV’s local tumor efficacy [31]. CPMV used for ISV upregulates immunostimulatory cytokines including IL-1β, IL-12, interferon (IFN)-γ, chemokine ligand 3, macrophage inflammatory protein-2, and granulocyte-macrophage colony-stimulating factor as well as suppressing IL-10 and transforming growth factor β [11,30,41]. These changes in intratumoral cytokines are generated by the changed phenotype of intratumoral myeloid cells and also mediate further activation, repolarization and recruitment of macrophages, DCs and neutrophils with an effector anti-tumor phenotype [41]. CPMV-ISV treatment significantly improves effector and memory CD4^+^ and CD8^+^ T cell responses and promotes systemic tumor-specific cytotoxic CD8^+^ T cell activity [41]. CPMV-based ISV treatment efficacy has been shown in mouse models of ovarian, breast, colon cancer, glioma, and melanoma [11,30,42–45] as well as in companion dogs with spontaneous tumors of multiple types [46]. CPMV, as a prophylactic and therapeutic immunotherapy can be used to target S100A9, a calcium-binding protein and suppressor of the tumor microenvironment, preventing manifestation of lung metastasis [47].

In vitro stimulation of CD14^+^ human monocytes with CPMV resulted in the induction of HLA-DR, CD86, PD-L1, IL-15R, CXCL10, MIP-1a and MIP-1b. CPMV also caused activation of dendritic cells and monocyte-derived macrophages. These findings demonstrated that CPMV activates human monocytes via Syk signaling, endosomal acidification, and recognition by Toll-like Receptor (TLR) 7/8. These findings support the potential for CPMV ISV to be an effective immune-based approach in humans [48].

### Empty CPMV (eCPMV) based monotherapy

eCPMV is an RNA-free VLP that induces an antitumor response that requires Th1-associated cytokines IL-12 and INF-γ, adaptive immunity, and neutrophils for full effect [12]. eCPMV treatment was superior in direct comparison to high dose LPS, poly(I:C), and STING agonist [11]. Thus, the immunostimulatory effect does not require on TLR stimulation by RNA and eCPMV is recognized by MyD88-dependent TLR2 and TLR4 but not TLR7 [31]. However, recent studies clearly show that RNA-containing CPMV is more effective in treating local tumors since it does stimulate TLR7 and generate type I IFN [15]. The value of TLR7 and associated type I IFN in generating systemic antitumor immunity is not yet demonstrated.

### Papaya mosaic virus (PapMV) based monotherapy

Rod-shaped PapMV contains single stranded RNA (ssRNA) that is primarily responsible for inducing human peripheral blood mononuclear cells (PBMC) to secrete type I interferon alpha (IFNα), IL-6 and other pro-inflammatory cytokines and chemokines. Internalization and disassembly of PapMV nanoparticles into the endosome leads to the release of ssRNA that can activate TLR7 and/or 8 and induce a strong immune response [33]. Intra-tumoral administration of PapMV significantly prolonged survival and correlated with enhanced chemokine and proinflammatory cytokine production in the tumor and increased immune cell infiltration [10].

### PVX based monotherapy

Flexible rod-shaped PVX has been studied as an immunotherapeutic for ISV monotherapy. In the context of B16F10 melanoma, PVX -based ISV can delay tumor progression, and with chemotherapies can be amplified [23].

### Tobacco mosaic virus (TMV) based monotherapy

TMV and TMV-short can elicit potent antitumor immunity after intratumoral treatment of dermal melanoma via strong pro-inflammatory cytokines, primarily IL-6, and the recruitment of innate immune cells and T cells. The treatment slowed tumor growth and increased survival time. [30].

### Alfalfa mosaic virus (AMV) based monotherapy

AMV is mixture of two bacilliform and spherical phenotypes, which encapsulate the virus genome. We investigated AMV as ISV in 4T1 mouse breast cancer, which is a very difficult mouse cancer model to treat with immunotherapy because of its recruitment of exceptional numbers of suppressive myeloid cells. AMV induced a potent immune response, which significantly delayed growth of this very challenging model. Response was characterized by IFN-γ, INF-α, IL-6, and IL-12 cytokines, and infiltration of CD4^+^ and CD8^+^ T cells at the treated site [49].

## PVNP-based Cancer Immunotherapies Combined with Other Cancer Therapies

Single reagent immunotherapy (monotherapy) is prevalent using checkpoint blocking antibodies against PD-1 or PD-L1, however most patients do not respond to single-approach immunotherapy. Currently, even responding patients generally eventually develop resistance, disease recurrence, and many patients have the treatment limited by toxicity due to autoimmunity caused by systemic checkpoint blockade administration [50,51]. It is increasingly recognized that combining immune therapies is the next stage of cancer immune therapy, just as combining chemotherapies dominates cancer chemotherapy [51]. The multiple advantages of ISV noted above make this a prime candidate for combination with checkpoint blockade and other developing systemic immunotherapies for solid tumors.

PVNP ISV contributes to combinatorial immune therapy by expanding the pool of antitumor effector T cells. This expanded T cell pool could be combined with immune checkpoint therapy, radiation therapy and chemotherapy to reduce tumor burden, prolong survival, and support expanded tumor-specific immune memory (Figure 1C). For example, intratumoral injected CPMV in combination with selected T cell focused antibodies; PD-1 blocking antibodies, or agonistic OX40-specific antibodies, and myeloid cell-focused CD47-blocking antibodies activates and recruits innate immune cells, thereby reprogramming the immunosuppressive tumor microenvironment toward an immune-activated state [52–54]. Utilizing combination radiation therapy (RT) with immunostimulatory CPMV suggests that CPMV in combination with RT can turn an immunologically “cold” tumor (with low number of tumor infiltrating lymphocytes) into an immunologically “hot” tumor [50]. Combination of CPMV and RT was tested and had efficacy in companion dogs with melanoma [55]. PVX immunotherapy and doxorubicin chemotherapy are best when co-administered separately into the tumor, allowing each drug to act on their own, leading to potent antitumor effects [56]. CPMV based ISV combined with cyclophosphamide reduced breast cancer tumor burden and inhibits lung metastasis [57].

## Conclusions

Studies show that PVNPs with their inherent immunostimulatory nature are valuable reagents for *in situ* vaccination and can be utilized as nanocarriers of tumor antigens to generate strong and sustained anti-tumor immune response without need for additional adjuvants. With nanoengineering, future designs could fuse the tumor antigens into CP of PVNPs, thus providing a means of cost-effective manufacture. PVNPs can induce antitumor responses in tumor models when administrated into a TME as an *in situ* vaccine, which alters the tumor microenvironment to an antitumor state, generating large numbers of tumor-specific effector T cells that supports systemic antitumor immunity and immune memory. Furthermore, significant therapeutic efficacy with prolonged survival can potentially be achieved when PVNPs-ISV combine with chemotherapy, radiotherapy, other option immunotherapies. While the wild type viruses are the current focus of research, PVNP engineering strategies may improve functionality and associated efficacy. Preclinical capabilities of PVNPs indicate that some PVNPs are more suitable for tumor immunotherapies than others and understanding the mechanisms of immune activation and relevant complex differences between PVNPs will set the stage for successful clinical development of PVNPs as a platform for cancer immunotherapy.
